# Chronic Lactate Exposure Decreases Mitochondrial Function by Inhibition of Fatty Acid Uptake and Cardiolipin Alterations in Neonatal Rat Cardiomyocytes

**DOI:** 10.3389/fnut.2022.809485

**Published:** 2022-03-04

**Authors:** Iñigo San-Millan, Genevieve C. Sparagna, Hailey L. Chapman, Valerie L. Warkins, Kathryn C. Chatfield, Sydney R. Shuff, Janel L. Martinez, George A. Brooks

**Affiliations:** ^1^Department of Human Physiology and Nutrition, University of Colorado, Colorado Springs, CO, United States; ^2^Department of Medicine, Division of Endocrinology, Metabolism and Diabetes, University of Colorado Anschutz Medical Campus, Aurora, CO, United States; ^3^Department of Medicine, Division of Medical Oncology, University of Colorado Anschutz Medical Campus, Aurora, CO, United States; ^4^Department of Medicine, Division of Cardiology, University of Colorado Anschutz Medical Campus, Aurora, CO, United States; ^5^Department of Pediatrics, University of Colorado Anschutz Medical Campus, Aurora, CO, United States; ^6^Exercise Physiology Laboratory, Department of Integrative Biology, University of California, Berkeley, Berkeley, CA, United States

**Keywords:** lactate, mitochondrial dysfunction, fatty acid metabolism, metabolic flexibility, fatty acids transport

## Abstract

**Introduction:**

Lactate is an important signaling molecule with autocrine, paracrine and endocrine properties involved in multiple biological processes including regulation of gene expression and metabolism. Levels of lactate are increased chronically in diseases associated with cardiometabolic disease such as heart failure, type 2 diabetes, and cancer. Using neonatal ventricular myocytes, we tested the hypothesis that chronic lactate exposure could decrease the activity of cardiac mitochondria that could lead to metabolic inflexibility in the heart and other tissues.

**Methods:**

Neonatal rat ventricular myocytes (NRVMs) were treated for 48 h with 5, 10, or 20 mM lactate and CPT I and II activities were tested using radiolabelled assays. The molecular species profile of the major mitochondrial phospholipid, cardiolipin, was determined using electrospray ionization mass spectrometry along with reactive oxygen species (ROS) levels measured by Amplex Red and mitochondrial oxygen consumption using the Seahorse analyzer.

**Results:**

CPT I activity trended downward (*p* = 0.07) and CPT II activity significantly decreased with lactate exposure (*p* < 0.001). Cardiolipin molecular species containing four 18 carbon chains (72 carbons total) increased with lactate exposure, but species of other sizes decreased significantly. Furthermore, ROS production was strongly enhanced with lactate (*p* < 0.001) and mitochondrial ATP production and maximal respiration were both significantly down regulated with lactate exposure (*p* < 0.05 and *p* < 0.01 respectively).

**Conclusions:**

Chronic lactate exposure in cardiomyocytes leads to a decrease in fatty acid transport, alterations of cardiolipin remodeling, increases in ROS production and decreases in mitochondrial oxygen consumption that could have implications for both metabolic health and flexibility. The possibility that both intra-, or extracellular lactate levels play roles in cardiometabolic disease, heart failure, and other forms of metabolic inflexibility needs to be assessed *in vivo*.

## Introduction

Despite observations that lactate is a favored over glucose as energy substrate at the whole body (1–5) and tissues levels in skeletal muscle (2, 3), heart (6–8) and brain (9), and that in isolated mitochondrial preparations lactate is readily oxidized (10–12), it has been observed that ROS production has been observed in myocytes incubated with prolonged high lactate levels (13). Such procedures result in upregulation of hundreds of genes associated with adaption to physical exercise including those of the Mitochondrial Lactate Oxidation Complex mLOC (14). However, in aggregate such observations give rise to the idea that while acute, but intermittent lactate exposure as occurs in physical exercise is adaptive, prolonged cellular exposure to lactate, as occurs in chronic inflammatory diseases, may be maladaptive particularly with regard to fatty acid oxidation, leading to metabolic reprograming and disease state.

Fatty acids in their CoA form are transported across mitochondrial inner membranes by carnitine palmitoyltransferase I and II (CPT I and CPT II). Under postprandial conditions, especially after carbohydrate ingestion, Acetyl-CoA levels increase due increase glycolysis and pyruvate formation increasing the level of Malonyl-CoA, which, besides promoting fatty acid synthesis, also inhibits CPT I, and therefore, fatty acid oxidation (15, 16).

Cardiolipin is a structurally unique dimeric phospholipid localized in the inner mitochondrial membrane where it is required for optimal mitochondrial function and biogenesis (17, 18). Cardiolipin is known to provide essential structural and functional support to several proteins involved in mitochondrial bioenergetics (19). A loss of cardiolipin content, alterations in its acyl chain composition, and/or CL peroxidation have been associated with mitochondrial dysfunction in multiple tissues in a variety of pathological conditions, including ischemia, hypothyroidism, aging, and heart failure (17). Aberrations in cardiolipin molecular species are a primary causative factor in the cardio-skeletal myopathy known as Barth syndrome, which is accompanied by a decrease in fatty acid oxidation in Barth mice and hearts from Barth patients (20–22). These observations underscore the important role of the cardiolipin molecular species profile in regulation of fatty acid oxidation.

Unlike previous studies of acute lactate exposure, to our knowledge, this study is the first to determine the effects of chronic lactate exposure for 48 h (hr) in cultured neonatal heart cardiomyocytes. Neonatal rat cardiomyocytes are the most frequently used cell culture models for research in cardiomyocytes and have been recognized as a valid cell culture model (23). In mice, at the beginning of embryonic phase the mitochondrial reticulum is immature containing few cristae and no matrix. However, by day 13.5 (E13.5), mitochondria, ETC and OXPHOS activities are indistinguishable from those in the adult (24–26). From work of Lopaschuk and colleagues (27) we know that in cardiomyocytes during the embryonic phase, almost the entire ATP synthesis is obtained through glycolysis which is key in the growth and differentiation of any proliferating cells. At birth there is a significant switch from almost 100% glycolysis to OXPHOS where 44% of ATP is derived from glycolysis and the reminding 56% from OXPHOS derived from lactate oxidation (25%), fatty acid oxidation (13%) and pyruvate oxidation (18%). In the Neonatal phase (7-days postnatal), the shift is significantly pronounced where OXPHOS accounts for 95% of ATP synthesis derived from lactate oxidation (49%), fatty acid oxidation (41%) and pyruvate oxidation (5%). At 21 days postnatal fatty oxidation accounts for 80%, pyruvate oxidation for 12%, glycolysis for 7% and lactate for 1% (27).

In the study herein, we show that in neonatal rat ventricular myocytes, chronic (continuous 48 Hr) exposure to high lactate decreases the activity of CPT II, and to a lesser extent, CPT I. The cardiolipin profile is also altered by lactate leading to an increase in species having 72 carbon fatty acyl chains. Furthermore, we show that lactate exposure increases reactive oxidative species (ROS) which have been linked to mitochondrial damage and dysfunction (28, 29), and lastly, chronic lactate exposure downregulates mitochondrial oxygen coupling efficiency and consumption rate.

Hence, in addition to the known inhibition of fatty acyl transport into the mitochondria by the malonyl-CoA mechanism (30), we sought to interrogate the hypothesis that there existed another mechanism regulating mitochondrial fatty acid uptake. Specifically, in extension of previous research (13) we hypothesized that chronic lactate exposure effects metabolic rate and energy substrate partitioning by downregulating activities of CPT I and II and causing structural changes in the cardiolipin scaffold. These results could be relevant to metabolic dysfunction in cardiometabolic disease (CMD), heart failure, and other forms of metabolic inflexibility.

## Materials and Methods

### Chemicals and Reagents

All chemicals were from Sigma Aldrich (St. Louis, MO, US) unless otherwise stated. A BCA protein assay (ThermoFisher, Waltham, MA) was used for protein quantification.

### Neonatal Rat Ventricular Myocyte (NRVM) Treatment

Neonatal rat ventricular myocytes (NRVMs) were isolated from the ventricles of 1- to 2-day–old Sprague Dawley rats (Charles River, Wilmington, MA US) by enzymatic digestions as previously described (31). Cells were plated for 16 hr in MEM-Hanks Salts medium with 5% fetal bovine calf serum including 1 g/L of glucose, 0.2X Penicillin G, and 2 ug/mL Vitamin B12. After 16 hr, Cells were then washed with Minimum Essential Medium (MEM)-Hanks Salts medium with L-glutamine (Gibco) containing transferrin (2 ug/mL), bovine serum albumin (0.1%), insulin (2 ug/mL), bromodeoxyuridine (10 ug/mL), HEPES (0.2M), Penicillin G (0.2X) and Vitamin B12 (2 ug/mL) with 0 mM, 5, 10, or 20 mM L-lactate treatments added. Cells were harvested or measurements taken after 48 hr of lactate treatment. All animal protocols are in accordance with Public Health Service Animal Welfare Assurance, ID A3269-01, and approved by the University of Colorado, Denver—Animal Care and Use Committee.

### Seahorse Assay

VMs were plated on a gelatin coated Seahorse 96-well plate at a density of 50,000 cells/well. Lactate was added after 24 h and mitochondrial function assessed by the Seahorse after 48 hr of lactate treatment in medium containing 1 mM of pyruvate, 2 mM of glutamate and 10 mM of glucose according to manufacturer suggestions. The Mito Stress Test kit was used with a XFe96 Seahorse Analyzer (Agilent, Santa Clara, CA US). Three separate NRVM preparations were used to collect Seahorse data. After the Seahorse assay, differences in live cell density for each well were normalized by incubating with the dye CyQuant (ThermoFisher, Waltham, MA, US) to image live cells, incubating for an hr and imaging with a fluorescent plate reader (iD5, Molecular Devices) according to manufacturer directions.

### Reactive Oxygen Species (ROS) Measurements

Amplex™ UltraRed Reagent (Life technologies) was used to measure superoxide plus hydrogen peroxide in cells. Briefly, NRVMs were plated on black 96-well microplates. Forty-eight hours post lactate treatment, cells were rinsed one time with phosphate-buffered saline (PBS). A solution containing Amplex Red dye (final concentration: 50 microM), hydrogen peroxide (0.0015% final) and superoxide dismutase (SOD, final concentration: 5 units/mL) was added to each well to a final volume of 200 microL and incubated in the dark at 37°C for 30 mins. Sample fluorescence was measured using a fluorescence platereader (iD5, Molecular Devices) at excitation and emission wavelengths of 540 and 600 nm, respectively. The cells were washed twice with 100 microL PBS and cell number was assessed using CyQuant cell proliferation assay kit according to the manufacturer's instructions (ThermoFisher). Sample fluorescence was measured using the same plate reader at excitation and emission wavelengths of 580 and 527 nm, respectively. Each condition was examined in 15 wells per plate in 4 different NRVM preparations leaving the edges free of cells. Data shown is from a single representative experiment.

### Carnitine Palmitoyltranferase I and II Activity Assays

Carnitine Palmitoyltranferase (CPT) I and CPT II activities were quantified in NRVMs using a ^14^C carnitine-based radioactivity assay previously described in detail elsewhere (32). The assay measures CPT I activity by permeabilizing the plasma membrane and measuring the production of palmitoylcarnitine from palmitoyl-CoA. The activity of CPT II was measured by permeabilizing the mitochondrial inner membrane and adding malonyl CoA to inhibit CPT I.

### Cardiolipin Quantification

Cardiolipin was quantified in NRVMs using previously published methods with liquid chromatography coupled to electrospray ionization mass spectrometry in an API 4000 mass spectrometer (Sciex, Framingham, MA) (33). After 48 hr of lactate treatment, cells were washed and scraped off plates using PBS. Lipids were extracted according to previously published methods with 1 mmol tetramyristoyl-cardiolipin as an internal standard (Avanti Polar Lipids, Alabaster, AL, US) (33, 34). Cardiolipin species were expressed as a percentage of the following cardiolipin species having a mass/charge (m/z) ratio of 1422, 1424, 1426, 1448. 1450. 1452, 1454, 1456, 1472, 1474, 1476, 1496, 1498, 1500. These percentages were summed for the species having side chains of 70 carbons (m/z 1422–1426), 72 carbons (m/z 1448–1456), 74 carbons (m/z 1472–1476) and 76 carbons (m/z 1496–1500). The fatty acyl side chains of the dominant form of these species are listed in Table 1.

**Table 1:** Number of each fatty acyl substituent on major species of individual cardiolipins.

**m/z**	**Total # Carbons**	**16:1**	**18:1**	**18:2**	**20:4**	**22:6**
		**palmitoleate**	**oleate**	**linoleate**	**arachidonate**	**docosahexanoate**
1422	**70**	1		3		
1424		1	1	2		
1426		1	2	1		
1448	**72**			4		
1450			1	3		
1452			2	2		
1454			3	1		
1456			4			
1472	**74**			3	1	
1474			1	2	1	
1476			2	1	1	
1496	**76**			3		1
1498			1	2		1
1500			2	1		1

*m/z, mass to charge ratio; fatty acid side chains are defined by number of carbons:number of double bonds*.

### Determination of Intracellular and Extracellular Lactate Concentrations

Media for 0, 5, 10, and 20 mM lactate was made and 1 ml saved at −80°C until further processing for measurement of *initial extracellular lactate*. The remainder of the media was added to three 60 mm plates per condition. After 48 h of incubation with lactate treatments, 1 mL of spent growth media was collected from each of three NRVM plates per condition and frozen at −80°C until further processing for measurement of *48 hr extracellular lactate*. Following aspiration of all spent media, cells were washed three times with cold PBS, 300 uL of cold PBS was added to each plate, and NRVMs were scraped and frozen at −80°C until further processing for measurement of *intracellular lactate*. Upon thawing, cells were kept on ice throughout the entire protocol. Cell suspensions were sonicated for 8 secs at 50% amplitude with a pencil-type sonicator (model 450, Branson, Danbury, CT) and 100 uL of each sample aliquoted into a clean microcentrifuge tube and protein concentrations determined using the Quick Start™ Bradford Colorimetric Protein Assay (#5000201, Bio-Rad, Hercules, CA) as per manufacturer's protocol. The remaining lysed-cell suspensions were spun at maximum speed in a microcentrifuge at 4°C for 15 mins (model PrismR, Labnet International, Edison, NJ). Following centrifugation, supernatants were transferred into new microcentrifuge tubes. Spent and initial growth media and intracellular (lysed-cell supernatant) lactate concentrations were determined via the L-lactate Colorimetric Assay Kit I from Eton Biosciences (#120001400A, San Diego, CA) as per the manufacturer's protocol. Colorimetry as measured via a Synergy 2 plate reader (BioTek, Winooski, VT) was employed to determine protein and lactate concentrations. Intracellular and spent media lactate concentrations were normalized to respective protein concentrations prior to further calculations/data analysis.

### Statistical Analyses

All data with the exception of reactive oxygen species measurements and intracellular/extracellular lactate measurements are graphed from multiple preparations on different days of primary cultured NRVMs and therefore for each preparation day, the data were normalized to untreated cells having a value of 1. Data analysis used Prism version 8.0 (GraphPad Software, La Jolla California USA). Treatment effects were analyzed using a 1-way ANOVA with corrections for multiple comparisons; the level of statistical confidence was set at *p* < 0.05. Data sets were tested for Gaussian distribution with D'Agostino & Pearson omnibus or the Shapiro-Wilk normality test. All data in this study conformed to a Gaussian distribution.

## Results

### Internal Concentrations of Lactate Reflect External Lactate in Neonatal Cardiomyocytes

**Figure 1:**
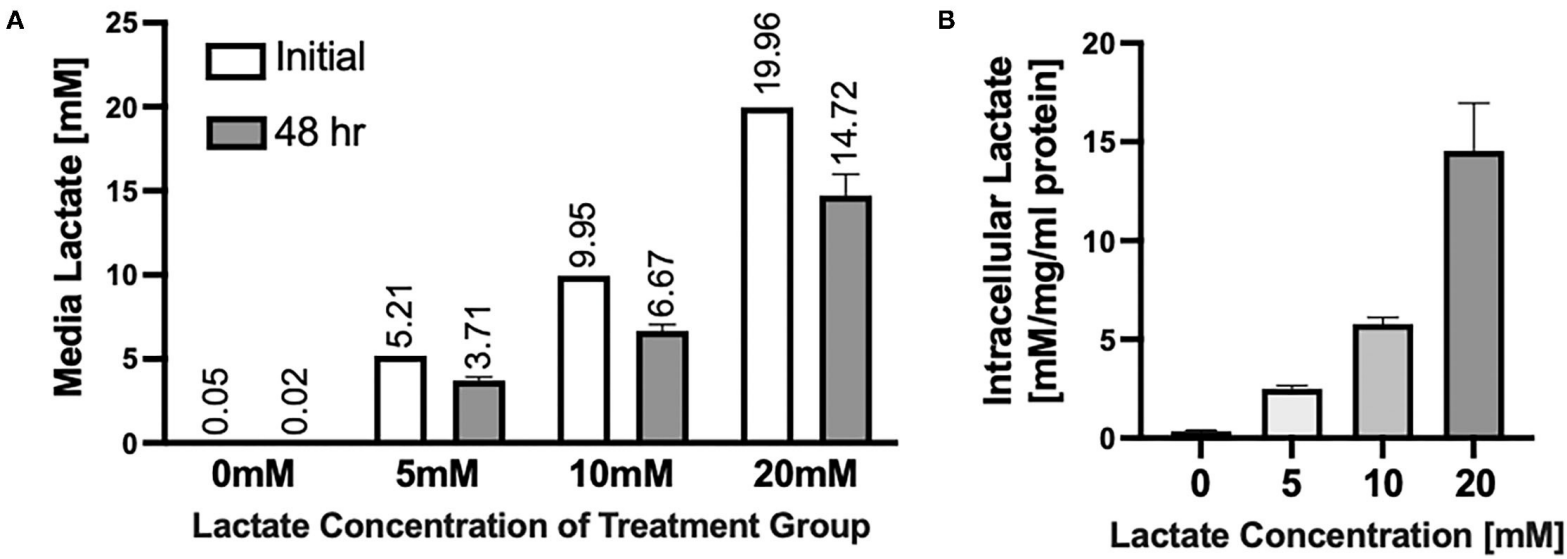
Uptake of Lactate into NRVMs **(A)** Extracellular media concentrations of lactate before (white bars) and after (gray bars) 48 h of lactate treatment. Numbers above bars indicate average of each measurement. **(B)** Intracellular lactate concentrations in NRVMs treated for 48 h with various concentrations of lactate. *N* = 3 plates, *n* = 1 solution (**A** white bars); error bars show standard error of the mean. *Intracellular lactate concentration is measured by mM of lactate per mg/ml of protein [mM/mg/ml protein).

Chronic lactate exposure results in the uptake of L-lactate in NRVMs and a resulting decrease in the media concentration of lactate (Figure 1A). Intracellular lactate in the NRVMs increases with increasing concentration of lactate treatment (Figure 1B). On one hand, NRVMs take lactate added to the media for ATP production. Further, in NRVMs, cytosolic glycolysis and lactate account for ~44 and ~25% respectively for ATP synthesis (27). Fetal bovine calf serum (FBS) contains 1 g/L of glucose needed for NRVMs survival. Due to their high glycolytic and lactate metabolism, it is expected that over time, intracellular lactate is increased. Moreover, it is known that lactate transport kinetics obey a gradient pattern through monocarboxylate transporters (MCT1/4) (35). Therefore, if there is a higher lactate concentration extracellularly compared to the intracellular one, the exportation of intracellular lactate could be compromise leading to intracellular lactate accumulation as could be the case of the 20 mM exposure.

### Lactate Exposure Decreases CPT I and II Activities

**Figure 2:**
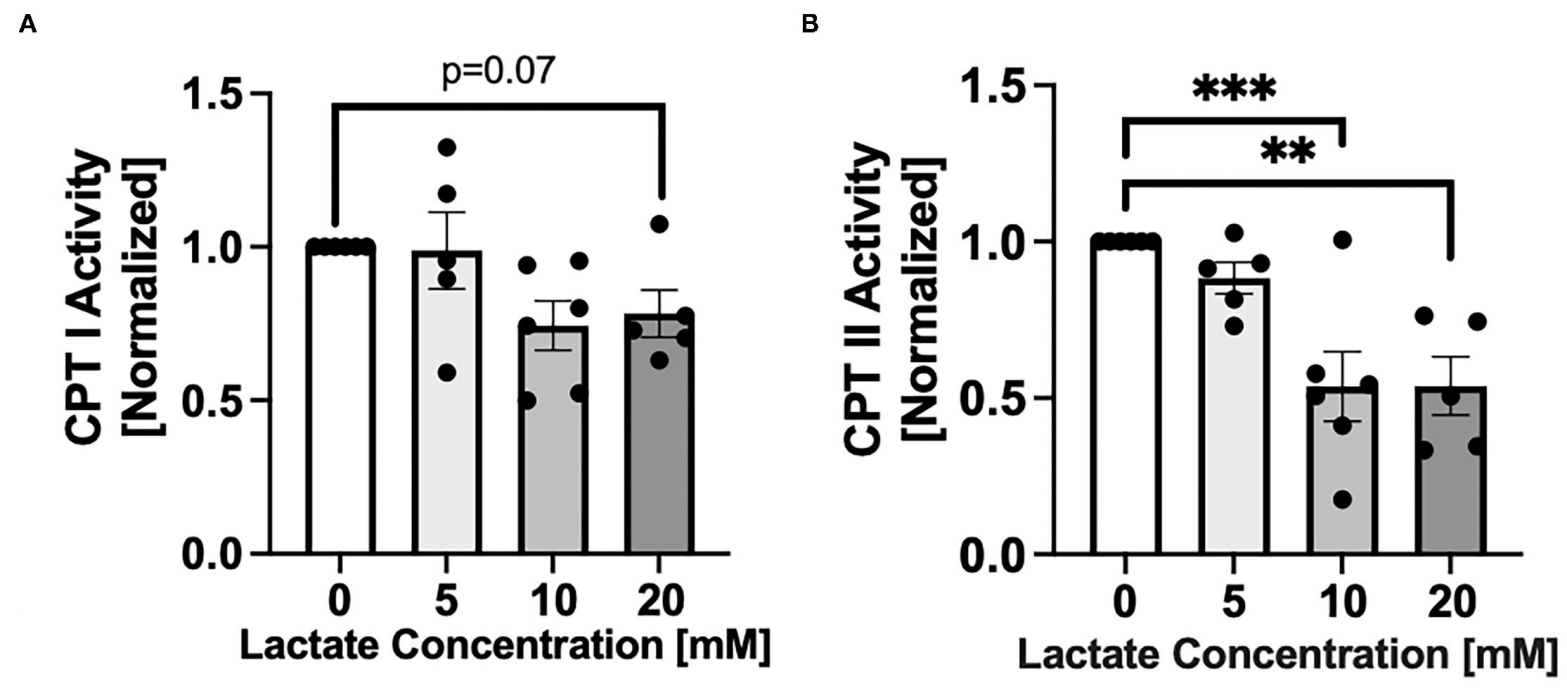
Carnitine palmitoyl transferase (CPT) activity with chronic lactate treatment. NRVMs were treated for 48 h with various concentrations of lactate and CPT I **(A)** or CPT II **(B)** assessed. Results are normalized to untreated cells with values for CPT I of 64.8 to 317.8 nmol/min/mg and for CPT II of 45.9 to 103.8 nmol/min/mg. *n* = average of 5–6 cell preparations; error bars show standard error of the mean, **p < 0.01, ***p < 0.001.

After 48 hr chronic exposure of NRVMs to 0, 5, 10, or 20 mM L-lactate, CPT activities decreased with increasing lactate concentration (Figure 2). There was a trend (*p* = 0.07) for a decrease of CPT I with 20 mM lactate exposure (Figure 2A). The effect was more pronounced for CPT II where 48 hr of lactate exposure of 10 and 20 mM significantly decreased CPT II activity (*p* < 0.001 *p* < 0.01, respectively, Figure 2B).

### Lactate Exposure Alters the Cardiolipin Profile

**Figure 3:**
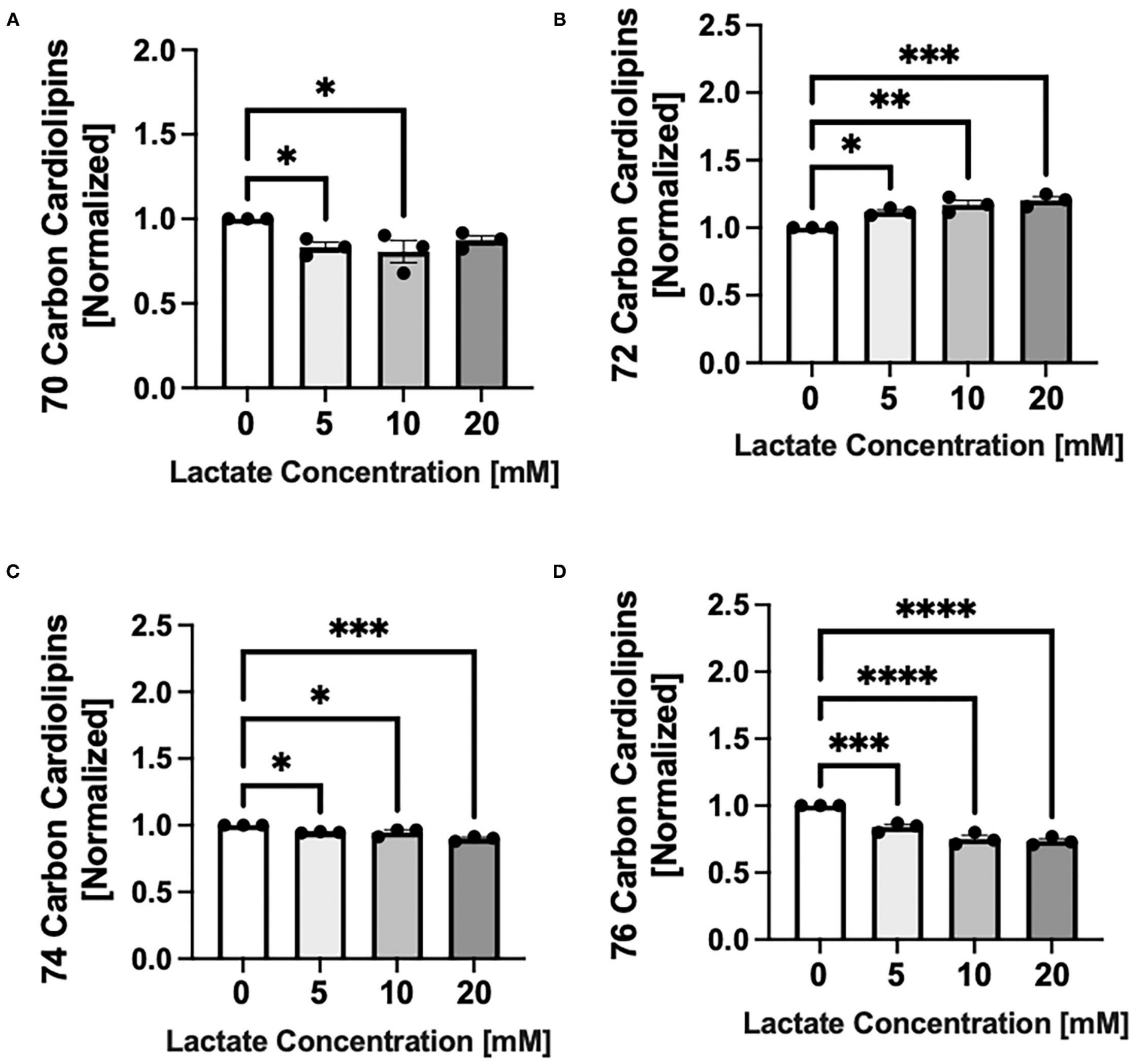
Cardiolipin molecular species profile with chronic lactate treatment. NRVMs were treated for 48 h with various concentrations of lactate and cardiolipin molecular profile assessed. Molecular species are expressed as a percent and size groups are normalized to the untreated sample from the same cell preparation day. Graphs show cardiolipin having total side chains lengths of **(A)** 70 carbons (untreated range from 9.5 to 10.0%), **(B)** 72 carbons (untreated range 40.2–49.3%), **(C)** 74 carbons (untreated range 27.2–29.2%) or **(D)** 76 carbons (untreated range 13.7–20.6%). *n* = average of 3 cell preparations; error bars show standard error of the mean, *p < 0.5, **p < 0.01, ***p < 0.001, ****p < 0.0001.

Chronic lactate exposure alters the cardiolipin molecular species profile in NRVMs in a concentration-related manner. Lactate causes a percentage increase in cardiolipin having a total side chain length of 72 carbons which are comprised of oleate and linoleate side chains *p* < 0.01 and *p* < 0.001 for 10 and 20mM respectively) (Figure 3B), but decreases in cardiolipins having either smaller (70 carbon) or longer (74 or 76 carbon side chains) (*p* < 0.05-p < 0.0001) (Figures 3A,C,D). Interestingly, there is no significant alterations in the ratio of L_4_CL (m/z 1448 having four linoleate side chains) to either O_4_CL (m/z 1456 having four oleates) or LO_3_CL (m/z 1454). This indicates that the side chain composition is not changing within the 72 carbon grouping (data not shown).

### Lactate Exposure Increases Reactive Oxygen Species Production

**Figure 4:**
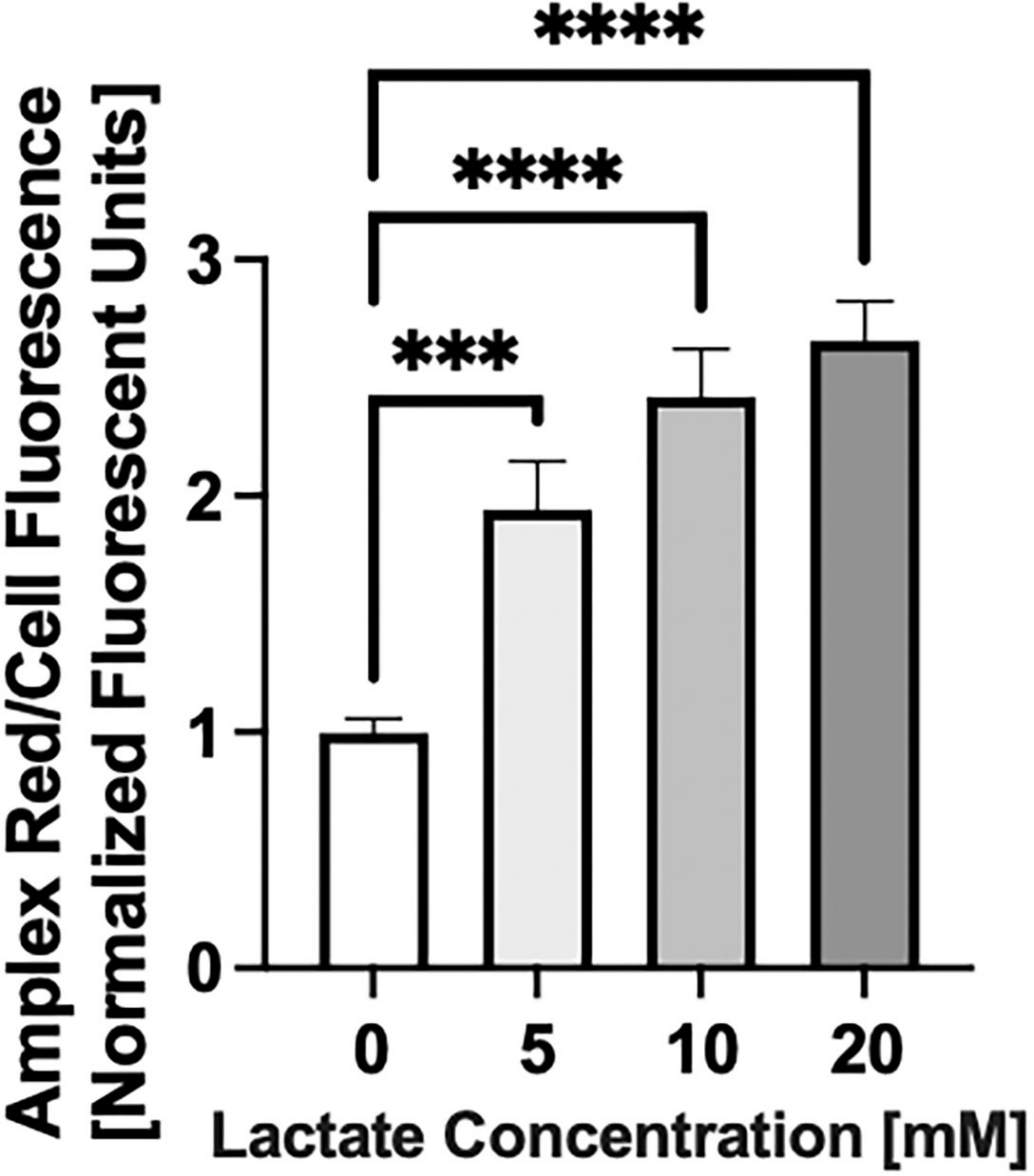
Reactive Oxygen Species (ROS) production with chronic lactate treatment. NRVMs were treated for 48 h with various concentrations of lactate and reactive oxygen species0 (total cellular superoxide plus hydrogen peroxide) assessed. Data shows a representative result from a single NRVM preparation. *N* = average of 15 wells on a 96-well plate. error bars show standard error of the mean, ***p < 0.001, ****p < 0.0001.

Chronic lactate exposure causes an increase in the level of mitochondrial reactive oxygen species (both superoxide and hydrogen peroxide) production in NRVMs (*p* < 0.5) as shown in Figure 4.

### Lactate Exposure Decreases Oxygen Consumption Rate and Maximal Respiration

**Figure 5:**
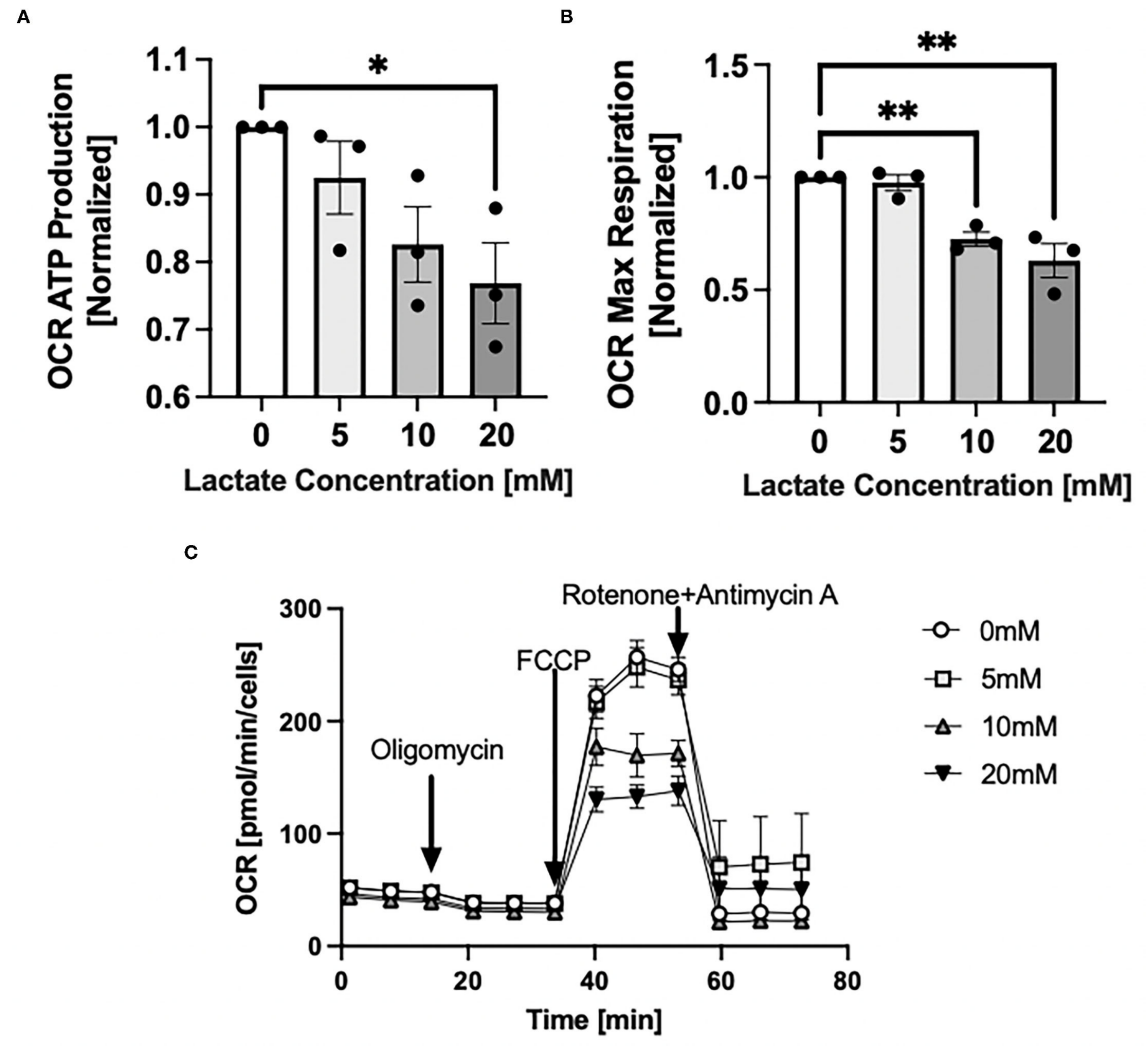
Mitochondrial oxygen consumption rate with chronic lactate treatment. NRVMs were treated for 48 h with various concentrations of lactate and oxygen consumption rate assessed using the Seahorse analyzer. **(A)** Rate of ATP production normalized to untreated cells (untreated range 8.5 to 16.3 pmol/min/cell), **(B)** Rate of maximal respiration normalized to untreated cells (untreated range 132.5 to 386.4 pmol/min/cell). **(C)** Seahorse trace for a representative NRVM preparation day. *n* = average of 3 cell preparations; error bars show standard error of the mean, *p < 0.05, **p < 0.01.

Through Seahorse system analysis, NRVMs with chronic exposure to lactate had decreased oxygen consumption rate (OCR) due to decreased ATP production (*p* < 0.05) (Figure 5A) and decreased maximal respiration (*p* < 0.05) (Figure 5B). Representative Seahorse Analyzer trace for one cell preparation day is shown in Figure 5C.

## Discussion

In this study we addressed the seemingly paradoxical roles of lactate in metabolism. Namely that intermittent exposure can be adaptive, while chronic exposure can be maladaptive. This concept has been long known in physiology and evidenced in this investigation (36). In sequence we discuss the chronic, maladaptive, and acute, adaptive effects of lactate exposure on metabolism. As a model system noted for metabolic flexibility, we employed a neonatal rat ventricular cardiomyocyte (NRVM) model to study the effects of chronic lactate.

As shown in Figure 1, lactate in the media decreases by ~30% as it is taken by cardiomyocytes and disposed of, likely through oxidation. Neonatal cardiomyocytes have already matured mitochondria capable of oxidizing lactate that enters mitochondria for oxidation via monocarboxylate transporter, MCT1 (37–39). While neonatal cardiomyocytes are quite efficient at oxidizing lactate (27), chronic hyper lactate elicits cellular disruptions in the activity of CPT1/2, cardiolipin, bioenergetics as well as cellular ROS production as shown herein.

### Effects of Chronic Lactate Exposure on CPT-I and –II

As a model system noted for the cellular oxidation of fatty acids, a major component of metabolic flexibility, we employed a neonatal rat ventricular myocyte (NRVM) model. Beyond autocrine and endocrine roles of lactate on regulation of lipolysis via covalent binding to GPR-81 (HCAR-1) (40), effects of lactatemia on depressing lipid oxidation as well as lipolysis have long been known (37). Furthermore, an impaired ability to clear pyruvate and lactate by oxidation (the enzymes of gluconeogenesis are not expressed in cardiomyocytes) leads to accumulation of malonyl-CoA, which is an inhibitor of CPT1 and mitochondrial fatty acid uptake and oxidation as previously shown by Saddik and colleagues (30). In the present study we show that lactate inhibits the activities of both CPT I and CPT II, especially the latter. Such effects would limit cellular lipid oxidation by downregulating mitochondrial uptake of carnitine-fatty acid derivatives. Those metabolic limitations are involved in the pathogenesis of insulin resistance and T2D (41, 42) and heart failure (43). We believe that our results showing effects of chronic lactate exposure on CPT I and II may explain in whole, or in part the relationship between chronic lactate exposure and metabolic inflexibility *in vivo*.

### Cardiolipin Effects of Chronic Lactate Exposure

In this study, results also show a unique perturbation of cardiolipin, the phospholipid that is highly integrated into the electron transport chain and other enzymes of fatty acid oxidation. These changes involve the enrichment of cardiolipins having oleate (18:1n9) or lineoleate (18:2n6) side chains, and a dramatic decrease in those cardiolipins having either smaller (palmitoleate, 16:1) or larger (arachidonate, 20:4n6 or docosahexaenoate, 22:6n3) fatty acyl side chains. This may be a direct effect of the inhibition of fatty acid transport via CPT since oleate and lineoleate are the most abundant unsaturated fatty acids and may already exist within the mitochondria to be put onto cardiolipin. Alterations to the cardiolipin molecular species profile of this sort have not been described before and further investigation is needed to determine the effect of these cardiolipin changes on mitochondrial function. However, the pattern of size shift in cardiolipin to four 18 carbon side chains is also seen in rat heart tissue during the developmental transition from the neonatal to the adult heart (Genevieve C. Sparagna and Kathryn C. Chatfield unpublished). What we do know from our studies with Barth Syndrome, where the cardiolipin profile is greatly altered, is that CPT function does not appear to be dependent on cardiolipin profile (22), making the cardiolipin alterations in this study not responsible for CPT activity decreases.

### ROS Generation and Chronic Lactate Exposure

Lactate exposure increases mitochondrial ROS generation, which may be a direct effect of the alteration of cardiolipin profile in the electron transport chain. There are multiple studies associating increased ROS with mitochondrial and cellular damage (28, 29, 44) and evidence supporting the involvement of ROS in the pathogenesis of T2D (45).

### Mitochondrial and Electron Transport Effects of Chronic Lactate Exposure

Lastly, while acute lactate exposure results in tightly coupled mitochondrial lactate oxidation (10, 12), prolonged exposure of NRVMs to lactate negatively affects mitochondrial respiratory control and coupling efficiency as well as the uncoupled maximal respiration rate. The mechanism by which chronic lactate exposure affects mitochondrial respiratory capacity and coupling efficiency may be associated with ROS production (Figure 4) or alterations in the cardiolipin profile (Figure 3). The response of NRVMs to chronic hyper-lactate exposure in culture may serve as a model of what happens *in vivo* where deficits in mitochondrial respiratory capacity may be behind the pathogenesis of multiple diseases including insulin-resistance, T2D and even cancer as mentioned *vide supra*. Decreases in mitochondrial function affected by acute and chronic lactate exposure may serve to decrease muscle mitochondrial fatty acid transport and oxidation *in vivo* resulting in metabolic inflexibility.

Further, decrease in maximal respiration might be attributed to cardiolipins modifications. The work done by Chatfield and Sparagna on mice and human heart show that in Barth Syndrome when cardiolipin is dramatically altered, there is decreased fatty acid utilization, increased ROS and decreased mitochondrial function (20) and (Genevieve C. Sparagna and Kathryn C. Chatfield unpublished). Thus, cardiolipin alterations can also contribute alone to changes in cellular bioenergetics. Moreover, herein we show that lactate plays an important role in regulating cardiolipin configuration.

### Lactate Metabolism in the Twenty-First Century

For nearly a century lactate has been considered as a “waste product” of anaerobic metabolism. However, we now know that lactate is formed under fully aerobic conditions, is a major energy source (1, 46–48), the major gluconeogenic precursor (46), a signaling molecule, a “lactormone” (37, 38) responsible for diverse actions such as gene expression (13, 49), and possibly a master regulator of carcinogenesis (50, 51). In the process of shuttling between sites of production and removal, lactate exerts profound effects on fat and carbohydrate (CHO) metabolism. Lactate is a preferred fuel over glucose in the beating heart (7, 52) working muscle (5, 46) and brain (9). Regarding fat metabolism, it is known that lactate binds G-protein coupled receptor (GPR81) that inhibits lipolysis in adipocytes (40, 53).

Lactate is oxidized *in vivo* in the mitochondrial reticulum via the mitochondrial lactate oxidation complex (mLOC) comprising a MCT1, its chaperone (CD147/Basigin), a mitochondrial LDH (mLDH), and cyclooxygenase enzyme (COX) (39, 54). It is well known that mitochondrial dysfunction is a hallmark in metabolic diseases like type 2 diabetes and cardiometabolic disease (CMD) characterized by poor fatty acid, carbohydrate oxidation and metabolic inflexibility (55–60). Thus, because lactate is a mitochondrial substrate, lactate oxidation is highly dependent on proper mitochondrial function as is demonstrated in individuals after exercise training (3, 61, 62). As early as 1962, Issekutz and colleagues observed the relationships between lactatemia and fatty acid oxidation (63). Recently, we have shown high inverse correlations (r > 0.9) between blood lactate levels and fatty acid oxidation rates. The inverse correlations hold on a wide range so human subjects ranging from those with metabolic syndrome to elite athletes (64).

Finally, the mechanisms by which chronic lactate exposure could elicit biological responses shown herein are not known and worth exploring in further studies. We believe that while acute, short-term lactate exposure, as occurs in physical exercise, is beneficial to skeletal muscle and other organ systems, chronic lactate exposure associated with lack of clearance could lead to metabolic dysregulation, and probably to disease. For example, Hashimoto et al. in the Brooks Lab showed that acute lactate exposure regulates 630 genes in mononucleated myotubes and striated L6 cells (13). Moreover, after studying MCF-7 breast cancer cells in culture we recently made a case that lactate accumulation from chronic aerobic lactate production (i.e., the Warburg Effect) in the absence of disposal, was an oncometabolite in cultured MCF-7 breast cancer cells (51). Although it may seem a paradox that acute lactate exposure, like in the case of exercise, could have beneficial effects, chronic lactate exposure, like in the case of cancer could be “detrimental.” The mechanisms behind acute vs. chronic lactate exposure must be further explored.

In summary, results of our study add to the current and emerging effects of lactate on acute and long-term regulation of metabolic rate and energy substrate partitioning. While hardly definitive or representative of metabolic regulation in humans and other mammals *in viv*o, a reasonable interpretation of the present results on cardiomyocytes is that they provide a partial explanation of the effect of lactatemia in downregulating mitochondrial lipid oxidation as might occur in heart failure and CMD (6, 43). On one hand, as reviewed above, lactate can be a preferred energy substrate when lactatemia is acute (37, 65, 66) including acute heart failure (67). However, on the other hand chronic intracellular and extracellular lactate exposure may negatively affect cellular bioenergetics by decreasing mitochondrial function, thus eliciting changes in fatty acid oxidation leading to metabolic inflexibility. Further research is needed to determine the mechanisms involved in decreased lactate clearance capacity that are involved in pathological conditions involving metabolic dysfunction in conditions and diseases associated with lactatemia.

## Data Availability Statement

The original contributions presented in the study are included in the article/supplementary material, further inquiries can be directed to the corresponding author/s.

## Author Contributions

IS-M, GB, and GS contributed to the hypothesis and experiments design as well as the preparation of the manuscript. GS, HC, VW, KC, SS, and JM contributed to the experiments and also to the manuscript. All authors contributed to the article and approved the submitted version.

## Funding

This study came from IS-M Cellular Metabolism Laboratory funds and supported by National Institutes of Health Grant R01 AG059715 to GB.

## Conflict of Interest

The authors declare that the research was conducted in the absence of any commercial or financial relationships that could be construed as a potential conflict of interest.

## Publisher's Note

All claims expressed in this article are solely those of the authors and do not necessarily represent those of their affiliated organizations, or those of the publisher, the editors and the reviewers. Any product that may be evaluated in this article, or claim that may be made by its manufacturer, is not guaranteed or endorsed by the publisher.

## References

[1] Mazzeo RS Brooks GA Schoeller DA Budinger TF. Disposal of blood [1-13C]lactate in humans during rest and exercise. J Appl Physiol. (1986) 60:232–41. 10.1152/jappl.1986.60.1.232 3080398

[2] Bergman BC Butterfield GE Wolfel EE Lopaschuk GD Casazza GA Horning MA. Muscle net glucose uptake and glucose kinetics after endurance training in men. Am J Physiol. (1999) 277:E81–92. 10.1152/ajpendo.1999.277.1.E81 10409131

[3] Bergman BC Wolfel EE Butterfield GE Lopaschuk GD Casazza GA Horning MA. Active muscle and whole body lactate kinetics after endurance training in men. J Appl Physiol. (1999) 87:1684–96. 10.1152/jappl.1999.87.5.1684 10562610

[4] Stanley WC Gertz EW Wisneski JA Morris DL Neese RA Brooks GA. Systemic lactate kinetics during graded exercise in man. Am J Physiol. (1985) 249:E595–602. 10.1152/ajpendo.1985.249.6.E595 4083344

[5] Miller BF Fattor JA Jacobs KA Horning MA Navazio F Lindinger MI. Lactate and glucose interactions during rest and exercise in men: effect of exogenous lactate infusion. J Physiol. (2002) 544:963–75. 10.1113/jphysiol.2002.027128 12411539 PMC2290635

[6] Brooks GA. Role of the Heart in Lactate Shuttling. Front Nutr. (2021) 8:663560. 10.3389/fnut.2021.663560 33968972 PMC8101701

[7] Gertz EW Wisneski JA Stanley WC Neese RA. Myocardial substrate utilization during exercise in humans. dual carbon-labeled carbohydrate isotope experiments. J Clin Invest. (1988) 82:2017–25. 10.1172/JCI113822 3198763 PMC442784

[8] Bergman BC Tsvetkova T Lowes B Wolfel EE. Myocardial glucose and lactate metabolism during rest and atrial pacing in humans. J Physiol. (2009) 587:2087–99. 10.1113/jphysiol.2008.168286 19289551 PMC2689346

[9] Glenn TC Martin NA Horning MA McArthur DL Hovda D Vespa PM. Lactate: brain fuel in human traumatic brain injury: a comparison to normal healthy control subjects. J Neurotrauma. (2015) 32:820–32. 10.1089/neu.2014.3483 25594628 PMC4530406

[10] Brooks GA Dubouchaud H Brown M Sicurello JP Butz CE. Role of mitochondrial lactate dehydrogenase and lactate oxidation in the intracellular lactate shuttle. Proc Natl Acad Sci U S A. (1999) 96:1129–34. 10.1073/pnas.96.3.1129 9927705 PMC15362

[11] Brandt RB Laux JE Spainhour SE Kline ES. Lactate dehydrogenase in rat mitochondria. Arch Biochem Biophys. (1987) 259:412–22. 10.1016/0003-9861(87)90507-8 3426237

[12] Young A Oldford C Mailloux RJ. Lactate dehydrogenase supports lactate oxidation in mitochondria isolated from different mouse tissues. Redox Biol. (2020) 28:101339. 10.1016/j.redox.2019.101339 31610469 PMC6812140

[13] Hashimoto T Hussien R Oommen S Gohil K Brooks GA. Lactate sensitive transcription factor network in L6 cells: activation of MCT1 and mitochondrial biogenesis. FASEB J. (2007) 21:2602–12. 10.1096/fj.07-8174com 17395833

[14] Hashimoto T Hussien R Cho HS Kaufer D Brooks GA. Evidence for the mitochondrial lactate oxidation complex in rat neurons: demonstration of an essential component of brain lactate shuttles. PLoS One. (2008) 3:e2915. 10.1371/journal.pone.0002915 18698340 PMC2488371

[15] Foster DW. Malonyl-CoA: the regulator of fatty acid synthesis and oxidation. J Clin Invest. (2012) 122:1958–9. 10.1172/JCI63967 22833869 PMC3366419

[16] McGarry JD Leatherman GF Foster DW. Carnitine palmitoyltransferase I. The site of inhibition of hepatic fatty acid oxidation by malonyl-CoA. J Biologic Chemistr. (1978) 253:4128–36. 10.1016/S0021-9258(17)34693-8 659409

[17] Chicco AJ Sparagna GC. Role of cardiolipin alterations in mitochondrial dysfunction and disease. Am J Physiol Cell Physiol. (2007) 292:C33–44. 10.1152/ajpcell.00243.2006 16899548

[18] Schlame M Rua D Greenberg ML. The biosynthesis and functional role of cardiolipin. Prog Lipid Res. (2000) 39:257–88. 10.1016/S0163-7827(00)00005-9 10799718

[19] Paradies G Paradies V De Benedictis V Ruggiero FM Petrosillo G. Functional role of cardiolipin in mitochondrial bioenergetics. Biochim Biophys Acta. (2014) 1837:408–17. 10.1016/j.bbabio.2013.10.006 24183692

[20] Le CH Benage LG Specht KS Li Puma LC Mulligan CM Heuberger AL. Tafazzin deficiency impairs CoA-dependent oxidative metabolism in cardiac mitochondria. J Biol Chem. (2020) 295:12485–97. 10.1074/jbc.RA119.011229 32665401 PMC7458807

[21] Cade WT Bohnert KL Peterson LR Patterson BW Bittel AJ Okunade AL. Blunted fat oxidation upon submaximal exercise is partially compensated by enhanced glucose metabolism in children, adolescents, and young adults with Barth syndrome. J Inherit Metab Dis. (2019) 42:480–93. 10.1002/jimd.12094 30924938 PMC6483838

[22] Chatfield KC Sparagna GC Spercht KS Witcomb LA Omar AK Wolfe LA. Mitochondrial morphology in metabolic diseases. J Inherit Dis Under Rev. 19:415–30. 10.1089/ars.2012.4779 22793999 PMC3700066

[23] Schwarzer M. Chapter 8 - Models to Investigate Cardiac Metabolism, In editors Schwarzer M Doenst T. The Scientist's Guide to Cardiac Metabolism (Boston: Academic Press) (2016), pp. 103–122.

[24] Hom JR Quintanilla RA Hoffman DL. The permeability transition pore controls cardiac mitochondrial maturation and myocyte differentiation. Dev Cell. (2011) 21:469–78. 10.1016/j.devcel.2011.08.008 21920313 PMC3175092

[25] Zhao Q Sun Q Zhou L Liu K Jiao K. Complex regulation of mitochondrial function during cardiac development. J Am Heart Assoc. (2019) 8:e012731. 10.1161/JAHA.119.012731 31215339 PMC6662350

[26] Mackler B Grace R Duncan HM. Studies of mitochondrial development during embryogenesis in the rat. Arch Biochem Biophys. (1971) 144:603–10. 10.1016/0003-9861(71)90367-5 4328160

[27] Lopaschuk GD Jaswal JS. Energy metabolic phenotype of the cardiomyocyte during development, differentiation, postnatal maturation. J Cardiovasc Pharmacol. (2010) 56:130–40. 10.1097/FJC.0b013e3181e74a14 20505524

[28] Cui H Kong Y Zhang H. Oxidative Stress, Mitochondrial Dysfunction, and Aging. J Signal Transduct. (2012) 2012:646354. 10.1155/2012/646354 21977319 PMC3184498

[29] Patergnani S Bouhamida E Leo S Pinton P Rimessi A. Mitochondrial oxidative stress and “mito-inflammation”: actors in the diseases. Biomedicines. (2021) 9:216. 10.3390/biomedicines9020216 33672477 PMC7923430

[30] Saddik M Gamble J Witters LA Lopaschuk GD. Acetyl-CoA carboxylase regulation of fatty acid oxidation in the heart. J Biol Chem. (1993) 268:25836–45. 10.1016/S0021-9258(19)74465-2 7902355

[31] Garcia AM Nakano SJ Karimpour-Fard A Nunley K Blain-Nelson P Stafford NM. Phosphodiesterase-5 is elevated in failing single ventricle myocardium and affects cardiomyocyte remodeling In Vitro. Circ Heart Fail. (2018) 11:e004571. 10.1161/CIRCHEARTFAILURE.117.004571 30354365 PMC6206883

[32] Yoon HR Hong YM Boriack RL Bennett MJ. Effect of L-carnitine supplementation on cardiac carnitine palmitoyltransferase activities and plasma carnitine concentrations in adriamycin-treated rats. Pediatr Res. (2003) 53:788–92. 10.1203/01.PDR.0000057988.62605.13 12621117

[33] Sparagna GC Johnson CA McCune SA Moore RL Murphy RC. Quantitation of cardiolipin molecular species in spontaneously hypertensive heart failure rats using electrospray ionization mass spectrometry. J Lipid Res. (2005) 46:1196–204. 10.1194/jlr.M500031-JLR200 15772420

[34] Bligh EG Dyer WJ. A rapid method of total lipid extraction and purification. Can J Biochem Physiol. (1959) 37:911–7. 10.1139/o59-099 13671378

[35] Halestrap AP. The monocarboxylate transporter family–Structure and functional characterization. IUBMB Life. (2012) 64:1–9. 10.1002/iub.573 22131303

[36] Selye H. Stress and the general adaptation syndrome. Br Med J. (1950) 1:1383–92. 10.1136/bmj.1.4667.1383 15426759 PMC2038162

[37] Brooks GA. The Science and Translation of Lactate Shuttle Theory. Cell Metab. (2018) 27:757–85. 10.1016/j.cmet.2018.03.008 29617642

[38] Brooks GA. Cell-cell and intracellular lactate shuttles. J Physiol. (2009) 587:5591–600. 10.1113/jphysiol.2009.178350 19805739 PMC2805372

[39] Hashimoto T Hussien R Brooks GA. Colocalization of MCT1, CD147, and LDH in mitochondrial inner membrane of L6 muscle cells: evidence of a mitochondrial lactate oxidation complex. Am J Physiol Endocrinol Metab. (2006) 290:E1237–44. 10.1152/ajpendo.00594.2005 16434551

[40] Liu C Wu J Zhu J Kuei C Yu J Shelton J. Lactate inhibits lipolysis in fat cells through activation of an orphan G-protein-coupled receptor, GPR81. J Biol Chem. (2009) 284:2811–22. 10.1074/jbc.M806409200 19047060

[41] Bergman BC Perreault L Strauss A Bacon S Kerege A Harrison K. Intramuscular triglyceride synthesis: importance in muscle lipid partitioning in humans. Am J Physiol Endocrinol Metab. (2018) 314:E152–64. 10.1152/ajpendo.00142.2017 28978544 PMC5866414

[42] Goodpaster BH Wolf D. Skeletal muscle lipid accumulation in obesity, insulin resistance, and type 2 diabetes. Pediatr Diabetes. (2004) 5:219–26. 10.1111/j.1399-543X.2004.00071.x 15601366

[43] Kawase T Toyofuku M Higashihara T Okubo Y Takahashi L Kagawa Y. Validation of lactate level as a predictor of early mortality in acute decompensated heart failure patients who entered intensive care unit. J Cardiol. (2015) 65:164–70. 10.1016/j.jjcc.2014.05.006 24970716

[44] Kowaltowski AJ Vercesi AE. Mitochondrial damage induced by conditions of oxidative stress. Free Radic Biol Med. (1999) 26:463–71. 10.1016/S0891-5849(98)00216-0 9895239

[45] Sivitz WI Yorek MA. Mitochondrial dysfunction in diabetes: from molecular mechanisms to functional significance and therapeutic opportunities. Antioxid Redox Signal. (2010) 12:537–77. 10.1089/ars.2009.2531 19650713 PMC2824521

[46] Bergman BC Butterfield GE Wolfel EE Casazza GA Lopaschuk GD Brooks GA. Evaluation of exercise and training on muscle lipid metabolism. Am J Physiol. (1999) 276:E106–17. 10.1152/ajpendo.1999.276.1.E106 9886956

[47] Brooks GA Wolfel EE Groves BM Bender PR Butterfield GE Cymerman A. Muscle accounts for glucose disposal but not blood lactate appearance during exercise after acclimatization to 4,300 m. J Appl Physiol. (1985) (1992) 72:2435-45. 10.1152/jappl.1992.72.6.2435 1629100

[48] Stanley WC Gertz EW Wisneski JA Neese RA Morris DL Brooks GA. Lactate extraction during net lactate release in legs of humans during exercise. J Appl Physiol. (1985) 60:1116-20. 10.1152/jappl.1986.60.4.1116 3084443

[49] Martinez-Outschoorn UE Prisco M Ertel A Tsirigos A Lin Z Pavlides S. Ketones and lactate increase cancer cell “stemness,” driving recurrence, metastasis and poor clinical outcome in breast cancer: achieving personalized medicine via Metabolo-Genomics. Cell Cycle. (2011) 10:1271–86. 10.4161/cc.10.8.15330 21512313 PMC3117136

[50] San-Millan I Brooks GA. Reexamining cancer metabolism: lactate production for carcinogenesis could be the purpose and explanation of the Warburg Effect. Carcinogenesis. (2017) 38:119–33. 10.1093/carcin/bgw127 27993896 PMC5862360

[51] San-Millan I Julian CG Matarazzo C Martinez J Brooks GA. Is Lactate an Oncometabolite? evidence supporting a role for lactate in the regulation of transcriptional activity of cancer-related genes in mcf7 breast cancer cells. Front Oncol. (2019) 9:1536. 10.3389/fonc.2019.01536 32010625 PMC6971189

[52] Stanley WC. Myocardial energy metabolism during ischemia and the mechanisms of metabolic therapies. J Cardiovasc Pharmacol Ther. (2004) S31–S45. 10.1177/107424840400900104 15378130

[53] Cai TQ Ren N Jin L Cheng K Kash S Chen R. Role of GPR81 in lactate-mediated reduction of adipose lipolysis. Biochem Biophys Res Commun. (2008) 377:987–91. 10.1016/j.bbrc.2008.10.088 18952058

[54] Hashimoto T Brooks GA. Mitochondrial lactate oxidation complex and an adaptive role for lactate production. Med Sci Sports Exerc. (2008) 40:486–94. 10.1249/MSS.0b013e31815fcb04 18379211

[55] Blaak EE van Aggel-Leijssen DP Wagenmakers AJ Saris WH van Baak MA. Impaired oxidation of plasma-derived fatty acids in type 2 diabetic subjects during moderate-intensity exercise. Diabetes. (2000) 49:2102–7. 10.2337/diabetes.49.12.2102 11118013

[56] Kelley DE Goodpaster B Wing RR Simoneau JA. Skeletal muscle fatty acid metabolism in association with insulin resistance, obesity, weight loss. Am J Physiol. (1999) 277:E1130–41. 10.1152/ajpendo.1999.277.6.E1130 10600804

[57] Kelley DE Simoneau JA. Impaired free fatty acid utilization by skeletal muscle in non-insulin-dependent diabetes mellitus. J Clin Invest. (1994) 94:2349–56. 10.1172/JCI117600 7989591 PMC330064

[58] McGarry JD. Banting lecture 2001: dysregulation of fatty acid metabolism in the etiology of type 2 diabetes. Diabetes. (2002) 51:7–18. 10.2337/diabetes.51.1.7 11756317

[59] Kelley DE Mandarino LJ. Fuel selection in human skeletal muscle in insulin resistance: a reexamination. Diabetes. (2000) 49:677–83. 10.2337/diabetes.49.5.677 10905472

[60] Kelley DE. Skeletal muscle fat oxidation: timing and flexibility are everything. J Clin Invest. (2005) 115:1699–702. 10.1172/JCI25758 16007246 PMC1159159

[61] Dubouchaud H Butterfield GE Wolfel EE Bergman BC Brooks GA. Endurance training, expression, and physiology of LDH, MCT1, and MCT4 in human skeletal muscle. Am J Physiol Endocrinol Metab. (2000) 278:E571–9. 10.1152/ajpendo.2000.278.4.E571 10751188

[62] Emhoff CA Messonnier LA Horning MA Fattor JA Carlson TJ Brooks GA. Direct and indirect lactate oxidation in trained and untrained men. J Appl Physiol. (1985) (2013) 115:829-38. 10.1152/japplphysiol.00538.2013 23788576 PMC8846964

[63] Issekutz BJ. Miller H. Plasma free fatty acids during exercise and the effect of lactic acid. Proc Soc Exp Biol Med. (1962) 110:237–9. 10.3181/00379727-110-274785877419

[64] San-Millan I Brooks GA. Assessment of metabolic flexibility by means of measuring blood lactate, fat, and carbohydrate oxidation responses to exercise in professional endurance athletes and less-fit individuals. Sports Med. (2018) 48:467–79. 10.1007/s40279-017-0751-x 28623613

[65] Brooks GA. Glycolytic end product and oxidative substrate during sustained exercise in mammals–the “lactate shuttle. Comparative Physiology and Biochemistry - Current Topics and Trends. Circulation. (1984) 84208–218. 10.1007/978-3-642-70610-3_15

[66] Brooks GA. Lactate production under fully aerobic conditions: the lactate shuttle during rest and exercise. Fed Proc. (1986) 45:2924–9. 3536591

[67] Nalos M Leverve X Huang S Weisbrodt L Parkin R Seppelt I. Half-molar sodium lactate infusion improves cardiac performance in acute heart failure: a pilot randomised controlled clinical trial. Crit Care. (2014) 18:R48. 10.1186/cc13793 24666826 PMC4057379

